# The Predictive Value of Eosinophil Cationic Protein and Lactate Dehydrogenase in Asthma: A Comparative Study of Serum Versus Sputum

**DOI:** 10.1097/WOX.0b013e3181b2fe64

**Published:** 2009-07-15

**Authors:** Amina Hamed Ahmad Al Obaidi, Abdul Ghani Mohamed Al Samarai, Jasim Al-Janabi, Abdulkareem Yahia

**Affiliations:** 1 Departments of Biochemistry and Medicine, College of Medicine, Tikrit, Iraq; 2 College of Pharmacy, Tikrit University, Tikrit, Iraq; 3 National Diabetic Centre, Baghdad

**Keywords:** ECP, asthma, LDH, serum, sputum

## Abstract

**Background:**

Serum and sputum eosinophil cationic protein (ECP) levels are correlated with asthma disease severity.

**Objective:**

To establish a diagnostic accuracy of ECP and lactate dehydrogenase (LDH) in serum (indirectly) and sputum (directly) as inflammatory markers in asthma.

**Patients and Methods:**

In a cross sectional study, 76 asthmatic patients with exacerbation were enrolled in the study. ECP was determined using enzyme linked immuno-assay.

**Results:**

Asthmatic patients compared with control subjects, had a significant higher levels of ECP and LDH in sputum. Both sputum and serum ECP and LDH were reduced significantly with prednisolone treatment. FEV1 was inversely correlated with sputum ECP, serum ECP, and sputum LDH. A significant positive correlation was noted between sputum ECP and sputum LDH. Serum LDH does not demonstrate any significant correlations with other variables. The area under receiver operating characteristic curve showed that sputum ECP (0.92) was a significantly an accurate marker more than serum ECP (0.81), sputum (0.80) LDH, and serum (0.65) LDH. Furthermore, the area under curve was lower for serum ECP (0.81) than that for sputum ECP (0.92). However, serum ECP (0.81) was more accurate marker than serum LDH (0.65).

**Conclusion:**

Serum and sputum ECP as eosinophilic inflammatory markers are associated with poor asthma control. Sputum ECP and LDH were significantly an accurate markers more than serum ECP and LDH.

## 

Previous studies have demonstrated that airway inflammation in bronchial asthma directly by bronchoscopic biopsies, BAL, or sputum examinations [1] or indirectly by measurements of biomarkers in peripheral blood [2]. The increase in blood eosinophils and serum eosinophil cationic protein (ECP) may be useful, indirect marker of airway inflammation in asthma [3]. Pizzichini et al [4] reported that proportion of eosinophils in sputum is a more accurate marker of asthmatic airway inflammation than the proportion of blood eosinophils or serum ECP. Recently, Jang et al [5] reported that the proportion of eosinophils in sputum have more accurate diagnostic markers of asthmatic airway inflammation than NO metabolites in sputum and blood in differentiating asthmatic patients from control subjects. However, all the reported studies deal with 1 to 3 parameters; this is the first study that evaluates ECP and lactate dehydrogenase (LDH) in blood versus sputum for the same patients. In the present study a comparative investigation of airway inflammation directly (sputum) and indirectly (serum) in asthma to establish diagnostic accuracy of ECP and LDH in serum and sputum was performed.

## Materials and method

### Patients

The subjects included in the study were outpatients from the Asthma and Allergy Center and Samara General Hospital outpatients Clinic. The diagnosis of asthma was performed by a specialist physician and was established according to the National Heart Blood and Lung Institute/World Health Organization (NHBLI/WHO) workshop on the Global Strategy for Asthma [6] Patients were excluded if they were smokers, if they had respiratory infection within the month preceding the study, a rheumatological illness, malignancy, diabetic, heart failure, history of venous embolisms, coronary heart disease, and liver or kidney diseases.

At enrolment, they all underwent full clinical examination, pulmonary function test, and blood sampling. Sputum samples were collected from patients when indicated. Normal volunteers were also enrolled in the study as a healthy control. None of them had any previous history of lung or allergic disease and were not using any medication. They had a normal lung function test (FEV1 > 80%) and negative skin allergy test. General stool examination was performed for all patients and control to exclude parasitic infections.

The sampling performed during the period from May 2004 to January 2007. All samples were collected at morning after an overnight fasting. A comparative investigation of airway inflammation directly (sputum) and indirectly (serum) in asthma to establish diagnostic accuracy of ECP and LDH in serum and sputum was performed. In a cross sectional study, 95 asthmatic patients (exacerbation) were enrolled in the study and only 76 of them completed the study. After entry to the study, all patients receive oral prednisolone 30 mg for 7 days, followed by reduction of 5 mg/d up to zero. Venous blood and sputum samples were collected at the time of entry to the study and after 2 weeks of treatment course for determination of sputum and serum ECP and LDH. The study was approved by the ethics committee of our college, and written consent was obtained from all participating subjects.

### Skin Prick Test

The skin prick tests were performed and evaluated in accordance with European Academy of Allergy and Clinical Immunology subcommittee on allergy standardization and skin tests using standard allergen panel (Stallergen, France). The panel included the common inhalant allergens.

### Sputum Collection

Sputum was induced only when it could not be produced spontaneously. Sputum induction was performed as described by Fahy et al [7].

### Determination of Serum ECP

Serum ECP was determined by ELISA kit (MBL MESCACUP ECP TEST) from Medical and Biologic Laboratories Co, LTD, Japan. The test was performed according to the instruction of manufacturer.

### Determination of LDH

LDH activity was measured in serum or sputum supernatant by an enzymatic rate method using pyruvate as substrate. The test was performed using a colorimetric method kit (Randox, UK) and the test it was performed according to instruction of the manufacturer.

### Statistical Analysis

The values are reported as mean ± SD and 95% CI. For statistical analysis between groups paired *t *test was used. Pearson test was used for correlation analysis. The diagnostic accuracy of sputum ECP, serum ECP, sputum LDH, and serum LDH were determined by generating a receiver operating characteristic (ROC) curve for each test. The levels of each marker were compared between the study groups and control group, using SPSS computer package. *P *values of < 0.05 were considered significant.

## Results

Asthmatic patients compared with control subjects, had significantly higher levels of ECP (Asthma, 751.8 μg/l; Control, 136.5 μg/l, *P *< 0.0001) and LDH (Asthma, 757.3 IU/l; Control, 25 IU/l, *P *< 0.0001) in sputum. Furthermore, serum ECP was significantly higher in asthmatic patients (46.1 μg/l) than in control (7.68 μg/l; *P *< 0.0001). In addition, serum LDH was significantly higher in asthmatic (453.9 IU/l) than in control subjects (126.3 IU/l, *P *< 0.0001) (Table 1).

**Table 1:** Markers of Airway Inflammation in Asthma: Sputum Versus Blood

Sample	Group	Variable	ECP *μ*g/l	LDH IU/l
Sputum	Asthma [76]	Mean	751.80	757.3
		SD	126.90	147.5
		95% CI	697.6-808	694.4-831.89
	Control [50]	Mean	136.50	25
		95% CI	119.2-153.96	22.65-27.35
		*P *value <	0.0001	0.0001
Serum	Asthma [76]	Mean	46.1	453.9
		SD	16.3	153.4
		95% CI	38.28-53.82	377.06-545.54
	Control [50]	Mean	7.68	126.3
		SD	5.63	57.2
		95% CI	6.08-9.68	110.04-142.56
		*P *value <	0.0001	0.0001

After treatment with prednisolone at dose of 30 mg/d for 7 days, followed by reduction of 5 mg/d up to zero. The sputum ECP was reduced significantly (*P *< 0.0001) from 751.8 μg/l before treatment to 313 μg/l after treatment. Furthermore, prednisolone reduced sputum LDH significantly from 757.3 IU/l before treatment to 276.8 IU/l after treatment. Serum ECP level was reduced significantly (*P *< 0.0001) from 46.1 μg/l before treatment to 16.42 μg/l after treatment. In addition, serum LDH was reduced significantly (*P *< 0.0001) from 453.9 IU/l to 219.9 IU/l after treatment (Table 2).

**Table 2:** Effect of Prednisolone on Airway Inflammation Markers Concentrations in Sputum and Serum of Asthmatic Patients

							Eosinophil Count			ECP/EO Count		
								
Variable	ECP Mean		μg/l SD	LDH Mean		IU/l SD	Mean		SD	Mean		SD
Sputum												
Pretreatment	751.8		126.9	757.32		147.5	0.41		0.25	1833		54
Posttreatment	313.0		119.8	276.8		82.1	0.23		0.34	1490		31
*P *value <		0.0001			0.0001			0.0001			0.0001	
Serum												
Pretreatment	46.1		16.3	453.9		153.4	479		174	0.096		0.043
Posttreatment	15.43		6.3	219.6		50.4	319		121	0.048		0.029
*P *value <		0.0001			0.0001			0.0001			0.0001	

ECP, eosinophil cationic protein; LDH, lactate dehydrogenase; EO, eosinophil.

Baseline sputum and serum values for ECP and LDH were significantly (*P *< 0.0001) higher than that for control subjects. However, the posttreatment values for serum and sputum values for all were still significantly higher than that of control. Thus, sputum and serum inflammatory markers were lower in normal subjects than in both untreated and treated patients.

The eosinophil counts and ECP/Eosinophil counts ratio were significantly in posttreatment as compared with baseline values for sputum and blood samples. These findings may suggest that determination of sputum ECP, LDH, Eosinophils count, and ECP/Eosinophil count ratio were with diagnostic accuracy for asthma as noninvasive markers.

**Figure 1:**
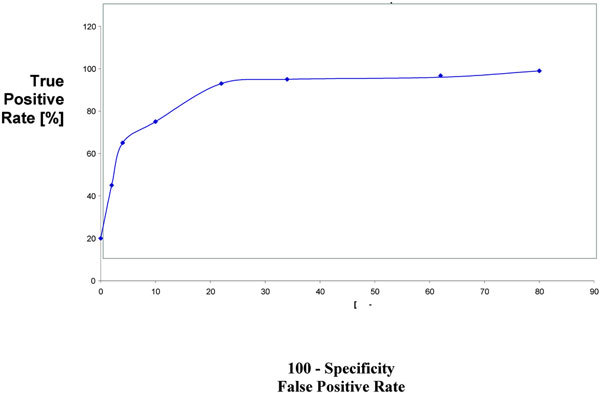
**ROC sputum ECP in asthmatic patients**.

**Figure 2:**
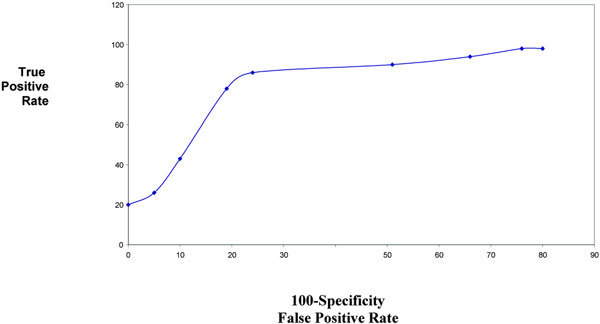
**ROC serum ECP in asthmatic patients**.

**Figure 3:**
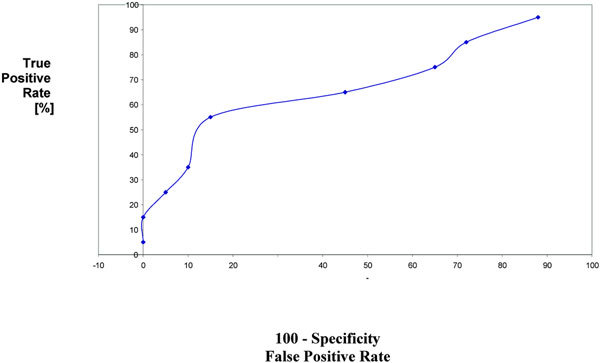
**ROC serum LDH in asthmatic patients**.

**Figure 4:**
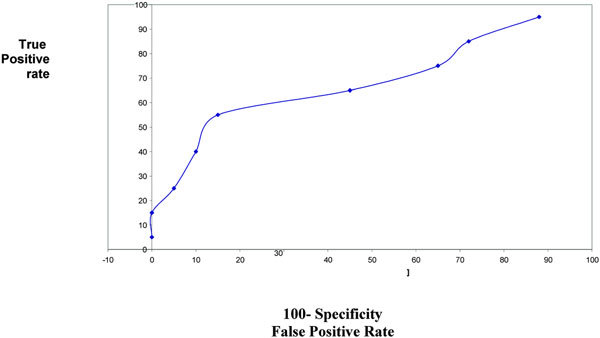
**ROC sputum LDH in asthmatic patients**.

The diagnostic accuracy of sputum and serum ECP and LDH was determined by ROC curve (Figures 1, 2, 3, 4). The area under ROC curve showed that sputum ECP (0.92) was significantly an accurate marker more than serum ECP (0.83), sputum LDH (0.80), and serum LDH (0.65). Furthermore, serum ECP with lower area under curve (0.83) than sputum ECP (0.92), however, serum ECP (0.83) was more an accurate marker than serum LDH (0.65).

## Discussion

In the present study sputum ECP and LDH were significantly increased in asthmatic patients as compared with control subjects. Furthermore, serum ECP and LDH were significantly higher in asthmatic than in controls subjects. This contributes that both (sputum and serum) can be used as direct and indirect markers of airway inflammation for sputum and serum ECP, respectively.

Holz et al [8] reported that sputum LDH was detected in asthmatic patients with high levels. Furthermore, Wark et al [9] reported that sputum LDH is elevated in acute asthma and it was as a marker of cell necrosis in asthma. An increase in airway LDH might arise from diverse sources, including: (a) rupture (necrosis) of airway and/or alveolar epithelial cells, alveolar macrophages, or other pulmonary cell types; (b) increased influx of plasma derived LDH through an air/blood barrier render more permeable by pulmonary injury (eg, edema); and (c) elevated plasma LDH concentration resulting in an increased rate of passage of LDH across the air/blood barrier of a normal lung [10] Necrosis of epithelial cells and granulocytes lead to the release of intracellular contents that may themselves be proinflammatory [11] One of these products is LDH that has been used to measure the extent of cell lyses [12] Thus, LDH may serve as indicator suggestive of disturbances of the cellular integrity induced by pathologic changes in asthma.

This is the first report that evaluates the LDH activity in sputum-versus-serum of asthmatic patients with comparison to healthy subjects. The levels of LDH were significantly higher in asthmatic than in control for both serum and sputum LDH. However, the magnitude of increase as compared with control was 30 times for sputum LDH, whereas it was around 4 times for serum. This may be a result of cell damage that leads to extracellular appearance of the LDH [12] and it may be more in the airway than in the blood.

In acute asthma there was an intense sputum Neutrophil influx and degranulation along with elevated ECP as this study indicated. Sputum neutrophils were associated with increased cell lyses measured by sputum LDH and increased sputum LDH and ECP were associated with a longer hospital stay [9] The majority of asthma exacerbation is to be associated with viral respiratory tract infection [13] Respiratory viruses infect bronchial epithelial cells, resulting in epithelial activation and increased Neutrophil recruitment and activation [14]. In addition, viral infection can cause extensive epithelial necrosis that is enhanced by neutrophils [15]. The high prevalence of viral infection in acute asthma was reported by different studies [9,13].

Wark's study [9] demonstrated that the mechanisms of virus induced exacerbation differ from those of noninfective acute asthma with increased neutrophilic inflammation. Those with infection had an intense Neutrophil infiltrate and degranulation that was not seen in noninfectious asthma. However, sputum ECP was elevated to a similar degree in both groups. Based on our results and that reported by Wark et al, it was suggested that sputum LDH was a good marker of asthma exacerbation and may be used to differentiate between infective and noninfective asthma. In fact, it is because of a widespread distribution of LDH in the body, serum LDH was abnormal in a host of disorders. It is released into the peripheral blood after cell death caused by, for example, ischemia, excess heat or cold, starvation, dehydration, injury, and exposure to bacterial toxins. Therefore, the total serum LDH is a highly sensitive but not specific test [12]. Furthermore, serum LDH is abnormal in a large number of disorders, its sputum level may be a noninvasive marker that differentiates asthmatics from normal subjects.

In the present study, the area under ROC curve showed that sputum ECP was significantly an accurate marker more than sputum LDH and serum ECP. Thus, ECP and LDH determination in sputum was significantly an accurate marker than their level in serum. Pizzichini et al [4] reported that the area under the ROC curve showed that sputum ECP was more sensitive than serum ECP in differentiation of patients with asthma from non asthmatic.

It is universally agreed that corticosteroids are the most effective asthma therapy that suppresses inflammation in asthmatic airways and they inhibit almost every aspect of the inflammatory process [16]. The serum ECP levels are high in asthma; however, they are affected by the administration of corticosteroids and decreased ahead of clinical improvement of asthma attacks [17]. Although, the sputum ECP was decreased concomitantly with clinical symptoms irrespective of the administration of corticosteroids [18]. Recently, Curric et al [19] reported that there was a significant reduction of sputum but not serum ECP that is in keeping with previous work that failed to show suppression after inhaled budesonide [20]. Furthermore, it may be considered that systemic indices of asthma control are more distinct indirect markers of inflammatory activity occurring predominantly in the bronchial mucosa [20]. In the present study ECP levels in sputum and serum were reduced significantly after treatment, this finding is consistent with that reported by Jang et al [21] concerning sputum ECP.

In fact, steroids cause inhibition of epithelial NO production, thus increasing the proliferation of T_H_1 cells, which produce interferon gamma. This in turn acts on T_H_2 cells to suppress the production of interleukin (IL)-4 and IL-5 [16]. Administration of oral prednisolone (30 mg/d) resulted in a fall of LDH and ECP in both serum and sputum. Basically, antiasthmatic drug treatment, including corticosteroids can attenuate inflammatory reaction by down-regulating the release of IL-5 and by falling NO metabolites and by suppression of eosinophil activity [21]. In the present study, LDH and ECP in sputum and serum were higher at baseline and declined significantly after prednisolone therapy, although the levels were still higher as compared with controls. These findings may reflect persistence of low levels of airway inflammation despite control of symptoms.

The usefulness and safety of the analysis of blood inflammatory markers in asthma are widely recognized. Recently, the analysis of induced sputum has been proposed as a safe, noninvasive tool in the study of airway inflammation in asthma. This study is conducted to test whether sputum analysis is more useful than blood analysis in the evaluation of airway inflammation in asthmatic patients. Thus, our findings suggest that examination of sputum can be used successfully to speculate airway inflammation noninvasively and to follow the effect (monitoring) of antiasthma treatment in stable and exacerbation asthma. Furthermore, this study indicated that sputum ECP, LDH, Eosinophil count, and ECP/Eosinophil count may be with predictive value to differentiate between stable and exacerbated asthma.

Airway inflammation has been considered as the primary cause to airway obstruction and hyperresponsiveness [22] The investigations into the mechanisms involved in the pathogenesis of asthma has been hampered by difficulties in gaining direct access to asthmatic airway for evaluating the inflammatory process. Until recently, it has not been possible in clinical practice to measure inflammation directly, and the presence or absence of airway inflammation has been assumed from symptoms, measurement of bronchial hyperresponsiveness (BHR) and the effect of treatment. Whereas, serum markers such as eosinophil count, ECP, NO metabolites were indirect measures of inflammation in asthma [23]. The introduction of a method of sputum analysis [24] that is a noninvasive safe tool for analysis, the cellular and biochemical components of the airway secretions has allowed the direct measurement of airway inflammation in asthma [7,24-27]. In fact, measurement of induced sputum has been shown to be a repeatable, valid, and feasible method for assessing eosinophilic airway inflammation [4,7,24,25,28]

Increased LDH activity at presentation with acute asthma was associated with more severe clinical disease. In fact, increased LDH activity, along with elevated sputum ECP are important predictors of more severe and protracted acute asthma [9]. It may also reflect a type of airway inflammation less responsive to corticosteroids. This indicates a potential role for sputum LDH measurement to predict clinical course in acute asthma.

The limitation of this study is the small-scale study population, which make the generalization of the finding is difficult. However, these study findings may encourage performing a large-scale comparative study between serum and sputum ECP and LDH.
